# Hospital Meetings

**Published:** 1906-06-16

**Authors:** 


### HOSPITAL MEETINGS.

### THE LONDON HOSPITAL.

At the quarterly Court of Governors of the London Hospital on June 6 the report of the committee for the past quarter was read. It contained several interesting paragraphs.

### Hospital Abuse and Economy.

Since the last meeting of the Quarterly Court the pressure upon the hospital beds had been maintained and the strain upon the out-patient department remained as great as ever. Referring to various reports as to the abuse of hospitals, the committee stated that they were fully alive to the danger of such abuse and were doing all in their power to prevent it. Every new out-patient was subjected to a rigorous examination as to his wages, his rent, and the number of persons dependent upon him. As a result of these inquiries many patients were refused treatment and referred either to a provident dispensary or to a local practitioner. The nature of the disease from which the patient was suffering was, of course, always taken into account. In order to keep a check upon abuse with regard to in-patients—which the committee thought was probably greatest among what is known as "request cases"—*i.e.* requests from outside doctors to various members of the staff to admit cases—an arrangement had been made, upon the suggestion of the visiting staff, that the names of all such patients should be given to the secretary and a form filled up by the outside local practitioner to the effect that he believed the case, for financial reasons, to be a right and fitting one for hospital treatment. Since the meeting of the last Quarterly Court, the annual report had been issued. It gave a *resumé* of the whole of the rebuilding scheme, now completed, having occupied eight years. In spite of the pressure of work, the cost per bed had for the last four years gradually decreased; dating from 1902 the figures were respectively £104, £103, £97, and £88. Persistent watch was kept that no waste or leakage of any kind occurred in any department, every pound subscribed to the hospital becoming a matter of careful consideration as to how best to spend it.

### Opsonic Treatment of Consumption and Lupus.

The work of the opsonic department was increasing and the committee had been compelled to appoint a second assistant. By making certain tests of the blood the patient's power of resistance to consumption could be measured. If the power of resistance was found to be low, means had been discovered to increase it, and this power was expected eventually to be the means by which this dread disease would be stamped out. Lupus was caused by the same microbe, in the one case (lupus) the bacillus attacking the skin and in the other (consumption) the lungs. Certain lupus patients resisted all treatment by the Finsen light, having had as many as 600 sittings without effect; on testing the blood of all these patients the resistance was found to be so low that the patient undoubtedly got reinfected as fast as he got cured by the light. Upon administering the new discovery, and thus raising the patient's power of resistance, in every case the patient healed quickly and after a very few sittings. This opsonic treatment, of course, increased the "cost per bed," obtained by dividing the ordinary expenditure by the number of beds. But the committee felt that they would be supported in making their chief aim not "cheapness," but "efficiency," and believed that the London Hospital as well as certain other hospitals—St. Mary's especially—was doing a national work in its endeavours to stamp out the "national scourge." The committee had found it necessary to appoint an extra medical and surgical registrar. There had hitherto been one of each, but so immensely had the work grown in the last ten years that the time had come when more help must be provided. The duties of a registrar in such a building as that were most important. Every disease was classified, every symptom noted and registered, so that when any patient was admitted the physician or surgeon in whose charge he was had a literature at his disposal fully describing every previous case of the kind. The Court would appreciate the immense value to science of such a register.

### The Maternity Department.

The "Marie Celeste" wards were doing thoroughly good work. Patients in their homes, within a mile radius of the hospital were treated by the hospital midwives, and the services of these nurses—not merely in treating the mother during her confinement, but in looking after the home, had been very much appreciated. Incidentally, too, the hospital was again doing work for the country in that it was preparing thirty-six midwives annually for the examinations of the Central Midwives Board, and thus helping to raise the standard of this most important profession. In connection with these wards the committee referred with hearty thanks to the gift from the Countess of Derby, who had undertaken to provide 100 bundles annually at her own expense of baby linen for the benefit of poor mothers confined in the wards, in order that when they and their babies left the hospital they should not be in want of necessaries.

### The Pathological Department.

The pathological department had received a great deal of attention during the quarter. The committee felt that it was not their duty to subscribe from the general funds of the hospital to the Pathological Institute; yet they considered the researches carried on there were of such inestimable advantage to all patients in the hospital that some endeavour should be made to put this department on a firm basis. The appeal made by the Chairman had resulted in the following subscriptions and donations : Lord Northcliffe and Mr. Hudson Kearley, M.P., £500 each per annum; Messrs. J. Keiller and Sons, £105; Mr. T. F. Blackwell, £52 10s.; Messrs. Travers and Son, £52 10s.; Messrs. Huntley Palmer and Son, £50; Mr. Robert Park Lyle, £10 10s.; and Messrs. Alex. Riddle and Company, £1 1s.—together, £1,271 11s. In the pathological department a most minute microscopical, chemical, and bacteriological research was made on cases which had died in the hospital; of course, only with the permission of the friends of the patients, who so appreciated the good results to other sufferers of these examinations that permission had been willingly given in more than eight cases out of ten for a post-mortem examination to be held, the greatest respect being always paid to the dead. Certain letters which had appeared in the public press on this matter had, however, given an entirely erroneous and unfair view of the action of the hospital authorities. It was no small point in favour of post-mortem examinations that nothing could be hidden or hushed up in a hospital. The physician while making his rounds was surrounded by hospital students; those students were present, if the illness ended disastrously, at the post-mortem examination, and had evidence which nothing could conceal as to whether the treatment was right or wrong. The special departments of the out-patient department—namely, the skin, Finsen light, aural, and ophthalmic have increased so rapidly that the committee had been obliged at the request of the visiting staff to render further assistance. Consequently, an extra house surgeon had been appointed to both the aural and ophthalmic wards with two clinical assistants for the ophthalmic O.P. department and one clinical assistant for the aural O.P. department.

In moving the adoption of the report, Mr. Holland said he thought they would realise that the amount of work done created a record at almost every Court. As to the expenditure, it had always been promised that when the buildings were finished they would be able to reduce their cost, and this had now been fulfilled. The greatest development that quarter was in the opsonic department, where a certain liquid or serum was injected into the blood and rendered the patient immune from consumption. The system was as yet only in the initial stages, but so far the results had been most satisfactory, especially where there were boils and spots, these being entirely cured. It was most helpful in the treatment of lupus in conjunction with the Finsen light, in which department they were still healing 100 patients a day.

The report was adopted.

### METROPOLITAN HOSPITAL.

The annual Court of Governors was held on June 7th. Mr. C. J. Thomas was in the chair, and in moving the adoption of the report stated that the hospital was doing more useful work at the present time than ever before. There had been an increase of both in-patients and out-patients, the former having increased from 1,220 to 1,341 and the latter from 36,167 to 38,712. They watched very carefully those persons who came as out-patients. Everyone who came to the hospital was treated the first time, and then their circumstances were inquired into, to find whether they were suitable patients for the hospital. If it was found that their circumstances would enable them to take the advice of the medical men in the neighbourhood and to pay for it they were recommended to go to them. Their great object at the hospital was to work with the medical men of the neighbourhood, and in no wise to supplant them. While they had every reason to believe they were doing more useful work each year to the neighbourhood, their receipts did not increase in the same proportion. Legacies—which were always a varying source of income—were £2,000 for last year, while in the previous year they had been £4,500. Their ordinary income was a little in excess of what it had been, but the hospital could not be carried on without a great deal of additional help, £14,000 having to be provided every year for administration and maintenance. The average cost of each in-patient had been reduced from £2 2s. 10d. to £1 18s. 3d. They were very glad to see this, as they were told theirs was an expensive hospital; but they claimed that the work done there was well done all round. They had been trying for some time to reduce expenses, and a sub-committee was then sitting to consider the matter. An electrical and $x$-ray department had been added, and Dr. A. C. Jordan had been put in charge of it. In conclusion he pleaded for an increase in annual subscriptions. The motion having been seconded and other resolutions passed, Lieut.-Colonel E. Montefiore proposed a vote of thanks to the chairman, and remarked that Mr. Thomas seemed to gloat over the increase in the number of in-patients. He considered it should be the aim and object of all hospitals not to increase their numbers, but to do all they could to treat those only who were seriously ill, and the trivial cases should be treated by the dispensaries. Their casualty department should be only for real accidents.

